# Abiotic Stress Alters the Nutritional, Metabolomic, and Glycomic Profiles of *Piper auritum* Kunth

**DOI:** 10.3390/foods14203543

**Published:** 2025-10-17

**Authors:** Adriana Chico-Peralta, Mar Villamiel, Paola Isabel Angulo-Bejarano, Aurea K. Ramírez-Jiménez

**Affiliations:** 1 Tecnologico de Monterrey, School of Engineering and Science, Av. Eugenio Garza Sada 2501 Sur, Monterrey C.P. 64849, NL, Mexico; adriana.chico@tec.mx (A.C.-P.); pangulobe@tec.mx (P.I.A.-B.); 2 Grupo de Química y Funcionalidad de Carbohidratos y Derivados, Instituto de Investigación en Ciencias de la Alimentación (CIAL) (CSIC-UAM) CEI (CSIC + UAM), Nicolás Cabrera, 9, Campus de la Universidad Autónoma de Madrid, 28049 Madrid, Spain; m.villamiel@csic.es

**Keywords:** *Piper auritum* K., Abiotic stressors, Low molecular weight carbohydrates, INFOGEST

## Abstract

Traditional diets based on diverse edible plants are increasingly threatened by climate change, which exposes crops to abiotic and biotic stressors such as drought, soil salinity, UV radiation, microorganisms, and insect herbivory. Understanding how these conditions influence both the nutritional and nutraceutical profiles, as well as the availability of key compounds, is essential to preserve their functional value. *Piper auritum* Kunth, used in Mexican gastronomy, was selected to assess two abiotic stress scenarios: drought stress (DS) and salicylic acid (SA) to simulate plant defense against pathogens and/or predators. We evaluated proximate composition, dietary fiber, total phenolics, total flavonoids, antioxidant capacity, low molecular weight carbohydrates (LMWCs), monomeric composition, and essential oil volatiles. Additionally, the simulated gastrointestinal digestion (INFOGEST) with an additional rat small-intestine extract (RSIE) revealed that both SA and DS shifted sugar distribution, especially for soluble and structural pools. SA treatment correlated with synthesis of secondary metabolites, particularly oxygenated and hydrocarbon terpenes. Both abiotic stressors modulated LMWC release during digestion, altering the distribution of sugars such as raffinose and galacturonic acid, with potential prebiotic implications. Essential oil analysis revealed stress-specific shifts in volatile composition, particularly in safrole, β-caryophyllene, and related terpenes. Beyond individual compound changes, the combined evaluation of composition, antioxidant properties, and volatile profile provides a comprehensive view of how abiotic stress can reshape the functional potential of *P. auritum*. To our knowledge, this is the first report on LMWC relative abundance across INFOGEST stages for a *quelite* species and on the integrated effect of DS and SA on its chemical profile. These findings highlight the importance of including compound release and functional traits, alongside chemical characterization, in future assessments of traditional plants under climate-related stress to safeguard their contribution to sustainable diets.

## 1. Introduction

The Food and Agriculture Organization has integrated the minimum dietary diversity metric into Sustainable Development Goal 2. This includes the metrics of consumption of a minimum of ten food groups linked to positive health effects, including dark green leafy vegetables. However, climate crisis-related anomalies pose a threat to the growth conditions for food crops, threatening diversity and security [1]. Climate directly influences plant growth by modifying structural elements and tissue integrity, for example, lowering foliar concentrations of macro- and micronutrients and reduction in above-ground biomass, probably due to reduced photosynthetic activity [1,2]. In response to environmental stressors, plants initiate secondary metabolic pathways which promote the synthesis of secondary metabolites to face the new environmental challenges [3]. These adverse conditions alter the nutritional profiles of various plants, especially those under human pressures [4,5]. *Piper auritum* Kunth (*P. auritum*), a traditional Mexican leafy green, exemplifies such species [6,7,8]. Its essential oil, characterized by compounds like safrole and phytol, contributes a unique aroma to traditional local dishes [9,10]. The phytochemical composition, proximate composition, dietary fiber, and metabolic pathways of bioactive compounds have been previously documented [11]. Nevertheless, the effects of environmental changes on its nutritional and nutraceutical aspects remain unexplored. Abiotic stressors, including drought and pests, significantly influence plant metabolic processes [11,12]. Carbohydrates possess multiple critical biological functions in edible plants. They maintain cell wall integrity and are often measured as dietary fiber through chemical or enzymatic methods [13]. Soluble sugars and oligosaccharides, typically found in extracellular regions, serve as energy reserves and signaling molecules [2,3]. Low molecular weight carbohydrates (LMWC) are particularly significant for both plant physiology and human nutrition. In the first case they act as energy storage, signaling molecules, osmotic pressure regulators, and biosynthetic precursors, while their role under physiological conditions may provide hints of their physiological role, such as blood sugar increase or potential prebiotic effects [1,5]. Traditional carbohydrate analyses often overlook the physiological significance of reducing sugars and dietary fiber [14]. Examining LMWC during digestion can reveal variations in their release and distribution, affecting glycemic responses [15,16]. Additionally, total monosaccharide profiles from acid hydrolysis offer insights into carbohydrate composition changes under stress [17,18]. Despite their significance, the effects of abiotic stress on carbohydrate composition in quelites remain unexamined. In vitro digestion models like INFOGEST recreate gastric and intestinal phases in controlled environments [15].

Gallego-Lobillo et al. (2021) [17] suggested that INFOGEST might not fully account for digestive enzyme roles in carbohydrate matrices and proposed incorporating RSIE for enhanced physiological relevance.

Considering the predominance of carbohydrates in plants, a comprehensive analytical approach is necessary. Recent developments in glycomics allow the exploration of monosaccharides, oligosaccharides, and glycans, elucidating structural complexity and nutritional implications. Suarez et al. (2025) [19] illustrated the benefits of merging glycomic analysis with chromatographic methods to identify stress-induced alterations in plant carbohydrates.

This study examined the nutritional and bioactive variations following a 120-day exposure to abiotic stressors. It employs a multicomponent analysis to evaluate the effects of abiotic stressors on *Piper auritum* Kunth, serving as an example of challenging environmental conditions that may have an impact on nutritional and phytochemical profiles in traditional edible plants.

## 2. Materials and Methods

### 2.1. Plant Management and Treatments

**Figure 1:**
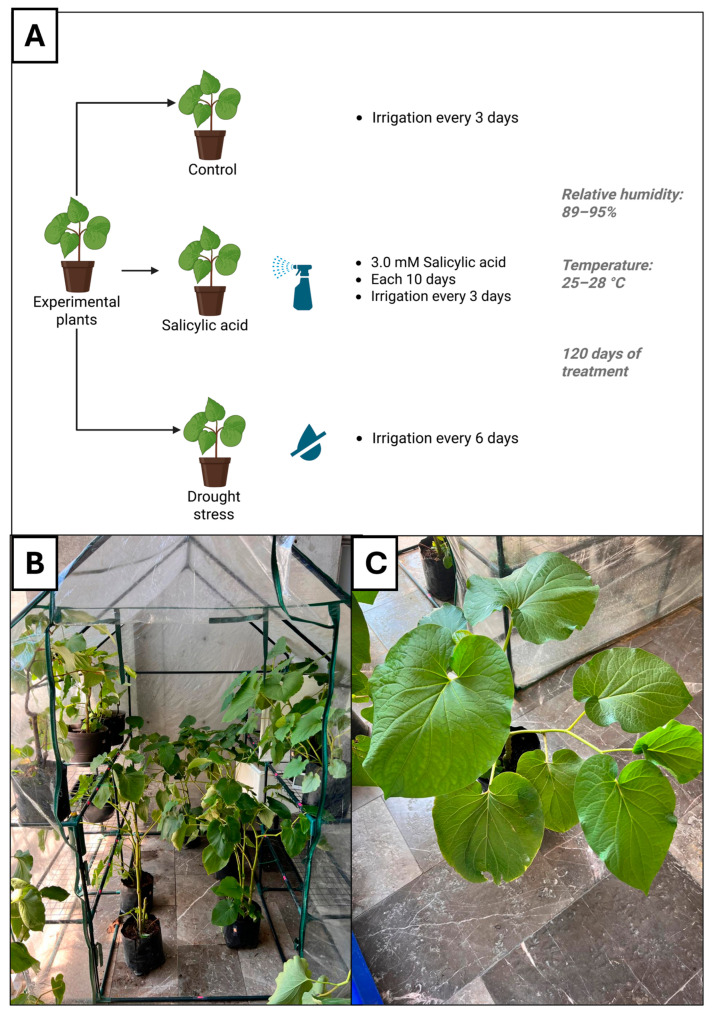
(**A**). Control group: plants were irrigated every three days with 500 mL of tap water; salicylic acid group: plants had the same irrigation frequency as the control group; drought stress group: plants were irrigated every six days with 500 mL of tap water. (**B**). The plants were stored in a warehouse with an average temperature of 27 °C, and an average humidity of 85%. Panel (**C**). Plant of *Piper auritum* Kunth. Created in BioRender.com.

Twenty specimens of *Piper auritum* Kunth, approximately one year old, were obtained from a local nursery in Querétaro, Querétaro, Mexico (20.5633° N, 100.3694° W; elevation: 1903 m; average annual precipitation: 570 mm). The plants were maintained in a greenhouse under 89–95% relative humidity and 25–28 °C (Figure 1A). Stem height and diameter were recorded for each plant. Taxonomic identification was confirmed at the ethnobotanical collection of the “Dr. Jerzy Rzedowski” Experimental Garden (QMEX) at the Autonomous University of Querétaro, where a voucher specimen was deposited under the accession number QMEX00006865 (Supplementary Figure S1). Leaves of *P. auritum* were dried at 40 °C for 12 h, ground to a fine powder, and stored at −80 °C until further use.

Plants were divided into control group (CT), salicylic acid group (SA), and drought stress group (DS). CT plants were irrigated every three days with 500 °C of tap water. SA was sprayed with 100 mL of salicylic acid 3 mM every 10 days. Its irrigation regime was the same as CT. Finally, DS plants were irrigated every 6 days. Humidity range was maintained between 89 and 95%, the temperature ranged from 25 to 28 °C. The experimental period extended for 120 days. Figure 1A summarizes the conditions of plants storage. Plants were maintained in a plastic shelter under shaded conditions, located adjacent to a sun-exposed area. To ensure homogeneous light exposure, pots were rotated weekly (Figure 1B,C).

### 2.2. Proximate Characterization

**Figure 2:**
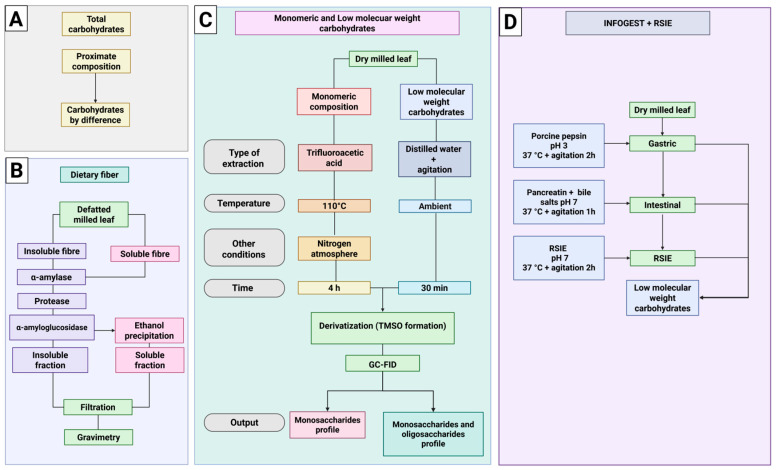
Workflow for the characterization of carbohydrates in *Piper auritum* leaves under control and treatment conditions. (**A**). Total carbohydrate content determined by difference from proximate composition; (**B**). Enzymatic–gravimetric method for dietary fiber, separating insoluble and soluble fractions; (**C**). Analysis of monosaccharides and LMWC: monomeric sugars released by trifluoroacetic acid hydrolysis under nitrogen atmosphere, and LMWC obtained by aqueous extraction; both derivatized and analyzed by GC-FID; (**D**). Sequential INFOGEST (gastric and intestinal) followed by rat small-intestinal extract (RSIE) incubation to monitor the relative abundance of LMWC across digestion stages. Created in BioRender.com.

The contents of protein (AOAC Method 920.87), lipids (AOAC Method 920.39), moisture (AOAC Method 925.10), and ash (AOAC Method 923.03) were determined following the official standardized procedures of AOAC International [20]. Carbohydrate content was calculated by difference (Figure 2A). Total dietary fiber (TDF), soluble dietary fiber (SDF), and insoluble dietary fiber (IDF) fractions, were determined according to AOAC Method 991.43. Total dietary fiber was analyzed with the Megazyme Assay Kit (Megazyme, Bray, Ireland) procedures followed the manufacturer’s instructions. A scheme of dietary fiber analysis is shown in Figure 2B.

### 2.3. Volatile Compounds

For GC-MS analysis, the compounds were extracted with methanol (1:10) as reported in our previous work [11]. The identification of volatile compounds was performed using gas chromatography coupled to an Agilent 5975C inter MSD. (GC-MS, Model 7890A, US81829145, Agilent Technologies, Santa Clara, CA, USA). The gas injector was maintained at 280 °C. One μL was injected into split mode using a Combi PAL (CTC Analytics, G6500-CTC) autosampler. The sample was separated using a DB-17ht column (30 m × 0.25 mm × 0.15 μm). Helium was used as a carrier gas at 3 mL/min flow rate. The oven temperature started at 50 °C and was programmed at 10 °C until 240 °C and remained 240 °C for 10 min. Spectra were recorded over the mass/charge (*m*/*z*) range 35 to 550 *m*/*z*. Peak identification was performed by comparing peak retention times to standards in the library NIST/EPA/NIH Mass Spectra Library v. 08 (NIST, USA).

### 2.4. Total Phenolic Compounds and Flavonoids

A methanolic extract was prepared by weighing 0.5 g of dry pulverized leaves and adding 10 mL of absolute methanol. The extract was protected from light and vortexed for 30 s, then placed in an ultrasonic bath (Cole-Parmer, Vernon Hills, IL, USA) for 20 min. Finally, the sample was centrifugated at 6000 RPM, and the supernatant was recovered. The total phenolic compounds analysis was performed according to the Folin–Ciocalteu method. The reaction was recorded using a MarkTM Spectrophotometer microplate reader (BIORAD, Tokyo, Japan) at 765 nm. The results were reported as milligrams equivalent to gallic acid per 100 g on dry weight basis (DW).

Total flavonoids were measured by following the procedure reported by Oomah et al. (2005) [21]. Briefly, 50 μL of the methanolic extract was mixed with 180 μL of methanol and 20 μL of a 10 g/L solution of 2-aminoethyldiphenylborate in a 96-well microtitration flat-bottom-plate (Corning, NY, USA). Absorbance was read at 404 nm in a microplate reader (BIORAD, Japan) using rutin as standard (0–50 μg/mL). The total flavonoid content was reported as milligrams of rutin equivalent (RE) per gram of sample (mg RE/100 g DW).

### 2.5. Carbohydrates Analysis

Regarding low molecular weight carbohydrates, a 150 mg sample was mixed with 250 μL of phenyl β-D-glucopyranoside (internal standard, 0.5 mg/mL) and 2250 μL of methanol (80% in water). The mixture was then stirred for 30 min and centrifuged at 13,000 rpm for 5 min. The resulting supernatant (1 mL) was collected, while the pellet was re-extracted under the same conditions (excluding the internal standard). The supernatants were then combined and dried.

Monomeric composition was obtained according to Muñoz-Almagro et al. (2017) [22]. Briefly, 30 mg of sample was mixed with 1.5 mL of 2 M trifluoroacetic acid (TFA) and under an inert environment using nitrogen. Samples were heated at 110 °C for 4 h followed by vacuum evaporation, addition of internal standard (400 μL of 0.5 mg/mL β-phenyl glucoside), re-evaporation, and derivatization.

Derivatization of both extracts were performed by adding 300 μL of hydroxylamine hydrochloride in pyridine (2.5%) to the dried sample, followed by incubation at 70 °C for 30 min with intermittent shaking. Subsequently, 300 μL of hexamethyldisilazane and 30 μL of concentrated trifluoroacetic acid were added, and the mixture was incubated at 50 °C for 30 min. The derivatized sample was then centrifuged at 10,000 rpm for 4 min, and the supernatant was transferred to a sealed vial for GC-FID analysis.

GC-FID analyses were conducted using an Agilent Technologies gas chromatograph (GC7890A) with a flame ionization detector (FID) and a Combi PAL (CTC Analytics, G6500-CTC) autosampler. The derived oxime-derived trimethylsilyl (TMSO) samples were separated utilizing a DB-5HT-fused silica capillary column (30 m × 0.32 mm × 0.10 μm) from J&W Scientific (Folsom, CA, USA). Nitrogen (N2) was used as a carrier gas at 1 mL/min flow rate. The detector temperature was maintained at 280 °C, and the oven temperature followed a programmed ramp from 150 °C to 380 °C at a rate of 10 °C/min. Sample injection was performed in split mode at a ratio of 1:20. Carbohydrate concentrations were calculated with the additional application of hydrolysis correction factors (HF) to account for incomplete release or degradation of monosaccharides during trifluoroacetic acid (TFA) hydrolysis. Carbohydrate quantification was performed using internal standardization. For each compound, the chromatographic peak area was compared with that of the internal standard. The calculation considered the known concentration of the internal standard and the specific response factor for each carbohydrate obtained from its calibration curve. Concentrations were expressed as milligrams per gram of sample (mg/g). To allow comparison across treatments and digestion stages, values were normalized to the total carbohydrate content quantified in each sample, and the contribution of each carbohydrate was reported as its relative abundance (%).

A scheme of differences among low molecular weight carbohydrates and monomeric composition is shown in Figure 2C.

### 2.6. In Vitro Digestion

The digestibility assessment of carbohydrates from *Piper auritum* Kunth leaves was conducted following the methodology of Gallego-Lobillo et al. (2021) [17]. Initially, 30 mg/mL of dried leaves were weighed into a vial containing distilled water. To simulate the stomach stage, the solution was mixed with an equal volume of simulated gastric fluid (2 mL of a porcine pepsin solution were added to obtain 2000 U/mL). Then, 5 μL of CaCl_2_ 0.3 M were added to the mixture, the pH was adjusted to 3, and then incubated in an orbital Thermomixer at 37 °C with continuous agitation. The sample was collected at 0 and 2 h, and the reaction was stopped by heating in boiling water for 5 min. For the intestinal stage, the solution of the gastric phase was mixed with an equal volume of simulated intestinal fluid (SIF) porcine pancreatin and bile salts were added to the digestion to achieve 100 U/mL and 10 mM, respectively. Then, 40 μL of CaCl_2_ 0.3 M was added and the pH was adjusted to 7.0. Digestion was carried out in an orbital Thermomixer at 37 °C with continuous agitation. An aliquot was taken at 1 h of reaction and inactivated in boiling water for 5 min. The gastric phase and intestinal phase lasted 2 and 1 h, respectively. Finally, 40 mg/mL of rat small intestine extract (RSIE) were added. The reaction was performed in an orbital Thermomixer during 2 h at 37 °C and 750 rpm. Aliquots were collected after 1 h, and the reaction was stopped by heating. Quantification of LMWC was performed as described in Section 2.5. A scheme of INFOGEST + RSIE is shown in Figure 2D.

### 2.7. Antioxidant Capacity by DPPH and ABTS

A standard Trolox calibration curve ranging from 20 to 800 µg/mL (0.08 to 3.2 mmol/L) was used for both ABTS and DPPH assays. Results were expressed as µmol Trolox equivalents per mg of sample (µmol Trolox/mg).

The 2,2-diphenyl-1-picrylhyydrazyl (DPPH) assay was conducted according to the procedure reported by [21]. Briefly, a DPPH solution (150 μM) was prepared in 80% (*v*/*v*) methanol. A total of 20 microliters of methanol extract were added to a well in a 96-well flat-bottom visible light plate containing 200 μL of DPPH solution. The absorbance was read at 520 nm after 0, 4, 10, 30, 60, and 90 min.

The antioxidant capacity values, expressed as Trolox equivalents (TEAC), were determined using 2,2′-azinobis-(3-ethylbenzothiazoline-6-sulfonic acid) (ABTS), following the method proposed by [23]. A volume of 20 µL of the methanolic extract obtained in Section 2.4 was mixed with 230 µL of ABTS radical solution. Absorbance was measured at 734 nm at room temperature.

### 2.8. Statistical Analysis

All measurements were conducted as independent experiments, in duplicate for monomeric composition, low molecular weight carbohydrates, and in vitro digestion assays, and in triplicate for total phenolics, total flavonoids, and proximate composition.

Data are presented as mean ± standard deviation. Pairwise comparisons between baseline and final periods were assessed using Student’s *t*-test, while differences among treatments were evaluated using one-way ANOVA followed by Tukey’s post hoc test. Statistical analyses were performed with JMP software (version 14.0.0). Statistical significance was set at *p* ≤ 0.05 for proximate composition, fiber content, and nutraceutical analyses, and at *p* ≤ 0.10 for monomeric composition, LMWC, and LMWC after in vitro digestion. Multivariate analyses were performed using MetaboAnalyst 6.0 (www.metaboanalyst.ca, accessed on 13 June 2025). Principal Component Analysis (PCA) was applied to visualize the clustering of samples based on the normalized relative abundance of volatile and semi-volatile compounds. Partial Least Squares Discriminant Analysis (PLS-DA) was conducted to identify metabolites contributing most to the separation among treatments, and Variable Importance in Projection (VIP) scores > 1 were considered discriminant. Hierarchical clustering heatmaps were generated from autoscaled data using Ward’s linkage method and Pearson correlation as the distance measure.

## 3. Results

### 3.1. Effect of Abiotic Stressors on Proximate Composition of P. auritum Leaves

To date, no formal studies have assessed the proximate composition of *P. auritum* leaves under abiotic stress. Table 1 displays the proximate composition before and after elicitation. Lipid content remained consistent across treatments: CT (7.08 ± 0.8–7.16 ± 1.32%), SA (5.25 ± 1.92–6.29 ± 1.79%), and DS (6.88 ± 1.28–6.14 ± 0.44%).

Previous reports indicate lipid contents of 20–80 mg/100 g FW in *P. auritum* and related quelites Pacheco-Hernández et al. (2022) [24] while recorded values of 4.65–11.04% in Oaxacan quelites, corroborating our data. Studies addressing stress effects on proximate composition are scarce; for example, Gu et al. (2020) [25] described downregulation of long-chain fatty acid biosynthesis in *Camellia sinensis* under drought without direct changes in lipid content. In contrast, no treatment reduced total lipid content here. Essential oils can behave differently, with water deficit enhancing yields in *Artemisia dracunculus* [26].

Protein content varied slightly across treatments, with a significant decline under SA by the end of the experiment (Table 1). This contrasts with Gu et al. (2020) [25] who found soluble protein increases in *C. sinensis* under drought, from 126 to 196 mg/g FW, far higher than the <5% typically reported for quelites [27]. Ash content was relatively stable but increased in CT after 120 days. Similar patterns have been reported in other plants: León-Sánchez et al. (2020) [3] noted that rainfall reduction decreased foliar nitrogen, phosphorus, and micronutrients. The higher ash in CT suggests that mineral accumulation was impaired by stress while continuing in unstressed leaves.

Total carbohydrates changed significantly only in CT after 120 days (Table 1). As structural polymers, carbohydrates dominate plant biomass through cellulose, lignin, and pectin (Kurzyna-Szklarek et al., 2022) [18]. León-Sánchez et al. (2020) [3] linked reduced rainfall to declines in dry biomass, particularly carbohydrate-rich tissues. In *C. sinensis*, drought suppressed lignin biosynthetic enzymes [22], supporting the view that DS limits wall carbohydrate synthesis. Pascual-Mendoza et al. (2023) [8] reported 46.14–76.73% carbohydrates (DW basis) in Oaxacan quelites, consistent with our findings.

Table 1 presents the soluble and insoluble dietary fiber levels in *P. auritum* Kunth leaves for control and treatment groups. The control group experienced a 23% increase in soluble fiber. No significant changes in insoluble fiber were noted, except for a 14% decrease in DS treatment.

The literature documents fiber contents in various quelites. Pascual-Mendoza et al. (2023) [8] indicated fiber content ranging from 10.1 to 53.6% per 100 g dry basis for these plants. Prior research indicated a total dietary fiber content of 27.14% *w*/*w* DW in *P. auritum* leaves, contrasting with 2% *w*/*w* FW reported by Pacheco-Hernández et al. (2023) [28]. Consumption of dietary fiber, particularly soluble fiber, may confer health benefits, although research on plant-derived carbohydrate advantages remains ongoing [26].

Insoluble fiber is recognized for its laxative properties and fermentation by colonic microbiota, contributing to increased fecal bulk. Gu et al. (2020) [25] noted downregulation of lignin-related enzymes under drought stress, corroborated by León-Sánchez et al. (2020) [3] who observed dry matter loss in leaves after prolonged drought. These findings may elucidate the absence of increases in soluble or insoluble fiber across tested groups.

### 3.2. Metabolomic Profile of Volatile Compounds

**Figure 3:**
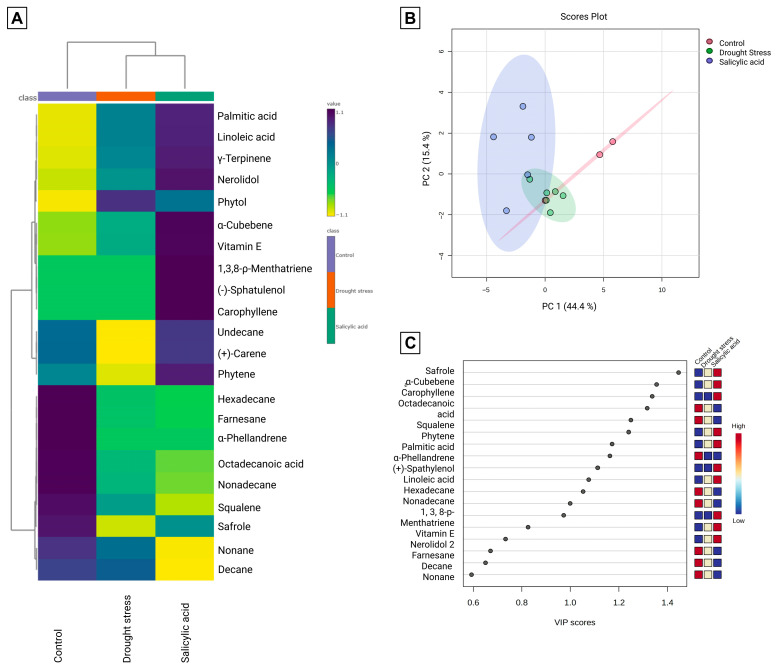
Multivariate analyses of volatile and semi-volatile compounds identified in plant samples at the final sampling period (control, drought stress, and salicylic acid treatments). (**A**) Hierarchical clustering heatmap generated from autoscaled data using Ward’s linkage method and Pearson correlation as the distance measure. Dark blue indicates values above the mean and yellow values below the mean, highlighting similarity patterns among treatments and metabolites. (**B**) Principal Component Analysis (PCA) score plot for PC1 (44.4%) and PC2 (15.4%), with ellipses representing the 95% confidence intervals. (**C**) Partial Least Squares Discriminant Analysis (PLS-DA) Variable Importance in Projection (VIP) score plot highlighting the metabolites contributing most to treatment separation (VIP > 1.0).

Figure 3 presents the clustered heatmap, PCA, and VIP score plot of volatile metabolites in CT, DS, and SA at the final period. The heatmap revealed clear treatment-specific patterns, while Figure 3A highlighted correlations between compounds. The volatile profile of *P. auritum* included safrole, fatty acids, and multiple terpenes, consistent with earlier reports on this species. However, the influence of abiotic stress on secondary metabolites has not been systematically studied.

Previous work in aromatic plants shows that stress enhances essential oil yields and compound diversity. Gorni et al. (2020) [29], for example, found increased terpene content in *Achillea millefolium* after SA elicitation, with PCA showing separating elicited plants from controls. A similar effect is evident in *P. auritum*, where PCA explained 59.8% of variance in the first two components (Figure 3B): CT clustered on positive PC1, SA on negative PC1, and DS near the origin, partially overlapping both. The VIP plot (Figure 3C) identified key metabolite-discriminant groups.

CT exhibited the highest safrole abundance, together with α-phellandrene, squalene, and several alkanes and fatty acids, while linoleic and palmitic acids were lowest. DS showed the lowest abundance of many essential oil compounds but contained the highest phytol levels. Some alkanes and sesquiterpenes increased under DS, together with γ-terpinene and vitamin E. In SA, sesquiterpenes, γ-terpinene, and vitamin E were the highest, while squalene and some alkanes decreased. Safrole in SA was lower than CT but higher than DS, indicating that stress influenced both terpene and phenylpropanoid pathways.

Terpenes are derived from acetyl-CoA and are stimulated by photosynthetic activity [30]. Their increase in SA-treated plants coincides with higher glucose at the final period, supporting a link between primary metabolism and terpene synthesis. Safrole, a phenylpropanoid derived from phenylalanine and the most abundant compound in *P. auritum* essential oil, decreased in DS. Drought limits photosynthesis and increases ROS, typically activating phenylpropanoid pathways [3], which may be the reason why safrole remained lowest in DS, suggesting that flux was diverted to other phenylpropanoids. SA, in contrast, appeared to enhance phenylpropanoid metabolism, yielding more phenolics and moderate safrole levels. These results may indicate that an adverse environment may not impact only *P. auritum* nutrient composition but also its aroma on food preparations. These secondary metabolite shifts suggest that terpenes and safrole modulation under stress not only reflect adaptive adjustments in secondary metabolism but could also directly influence the culinary and functional value of *P. auritum* as a traditional quelite, where aroma is central to its food use.

Squalene was most abundant in CT, lower in DS, and further reduced in SA. As a precursor for triterpenoid saponins, this may indicate conversion under stress. Indeed, drought has been associated with elevated saponins in *Glycyrrhiza glabra* [31]. Vitamin E content varied similarly: DS produced higher tocopherol levels, linked to increased phytol, consistent with its biosynthesis from phytol and homogentisate [32]. Tocopherols function in ROS detoxification and ABA regulation during drought. SA also promoted α-tocopherol accumulation together with higher terpene and sesquiterpene content, highlighting its role as a defense elicitor.

SA enhanced monoterpenes, sesquiterpenes, phenylpropanoids, and tocopherols, consistent with activation of defense metabolism. DS reduced safrole and several essential oil components but favored fatty acids, alkanes, and tocopherols, reflecting a shift toward structural stability and oxidative protection.

### 3.3. Bioactive Composition

**Figure 4:**
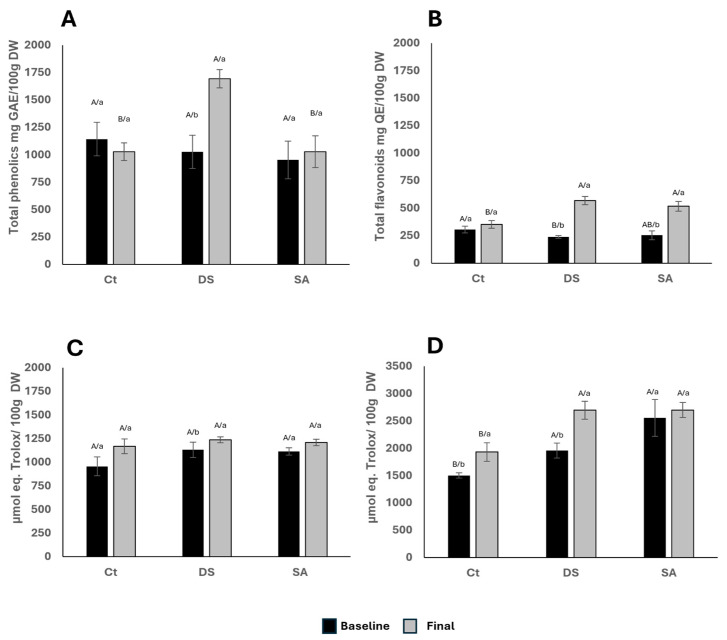
(**A**). Total phenolic compounds content expressed as gallic acid equivalents (GAE) by 100 g of dry weight (DW); (**B**). Total flavonoid content expressed as quercetin equivalents (QE) by 100 g DW. (**C**). Antioxidant activity by DPPH and (**D**). Antioxidant activity ABTS, both expressed as μmol eq. Trolox/100 g DW. Characters indicate comparisons between treatments (uppercase) using Tukey–Kramer test (α < 0.05) and time (lowercase) using t Student test (α < 0.05).

Figure 4A shows that DS increased total phenolic content (TPC) from 1026 to 1694 mg GAE/100 mg DW, a 65% rise compared with CT. Few studies have addressed elicitation in *P. auritum* [33] applied chitosan foliarly for one month, raising TPC by ~600%. Similarly, drought stress elevated phenolics in *Satureja hortensis* from 18.87 to 23.9 mg/g DW [32]. Higher TPC has also been reported in dried *P. auritum* sold in markets, likely reflecting collection and processing conditions [34].

Total flavonoids (TF), expressed as mg QE/100 mg DW, increased by 138% in DS and 103% in SA (Figure 4B). Fernández et al. (2021) [35] reported modest TF increases with chitosan, and their concentrations exceeded those found here. Chacón-Fuentes et al. (2017) [34] suggested that wild species accumulate more flavonoids than cultivated ones, highlighting environmental influences. SA has been shown to enhance flavonoid biosynthesis broadly; Stasińska-Jakubas et al. (2024) [36] reported increased epicatechin and quercetin in *Hypericum perforatum*, while Kianersi et al. (2020) [37] found higher rutin in *Capparis spinosa* with SA and methyl jasmonate. These patterns agree with the increases in SA and DS observed here. Our previous work confirmed that flavonoid biosynthesis pathways are constitutively active in *P. auritum* leaves [11].

### 3.4. Carbohydrates

When a food item is evaluated for its carbohydrates content, its analysis usually consists of reducing sugars content, dietary fiber, and total carbohydrate content. Although valuable, this information does not encompass other aspects impacting the physiological role of carbohydrates. Differences among physiological responses might provide hints of their role, such as blood sugar increase or potential prebiotic effects. In addition to nutritional aspects, the total monosaccharide profile obtained after a strong acid hydrolysis provides information about the very primary composition of a food item and its changes on it related to its environment. Since carbohydrates are the main component in edible plants, a broader analysis of their composition would contribute to predicting its physiological role and its most basic composition. Among the chromatographic techniques used to evaluate carbohydrates, gas chromatography with flame ionization detection (GC-FID) is suitable to detect sugars and oligosaccharides when these are derivatized before their analysis. The formation of trimethylsilyl oximes guarantees their interaction with the stationary phase. Additionally, FIDs are suitable for the determination of monosaccharide derivatives [18].

**Figure 5:**
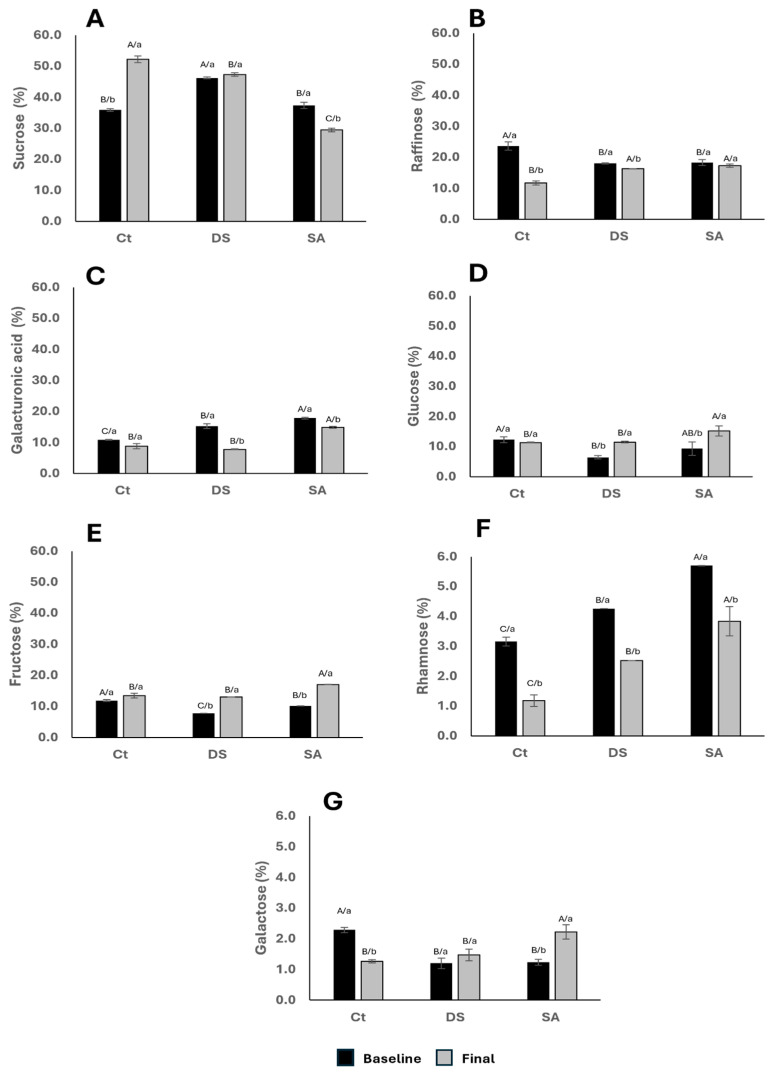
Low molecular weight carbohydrates composition. Relative content of (**A**). Sucrose; (**B**). Raffinose; (**C**). Galacturonic acid (**D**). Glucose; (**E**). Fructose; (**F**). Rhamnose; (**G**). Galactose. Characters indicate comparisons between treatments (uppercase) and time (lowercase), respectively, using Tukey–Kramer test (*p* < 0.10) and t Student test (*p* < 0.05), respectively.

Low molecular weight carbohydrates (LMWC) comprise monosaccharides, disaccharides, and oligosaccharides recovered by aqueous extraction and agitation. They occur in cytoplasmic and vacuolar pools, in extracellular spaces, or loosely associated with the cell wall, and contribute to osmoregulation, energy storage, signaling, and as precursors for secondary metabolism. Figure 5 summarizes ranges of their relative abundance for CT, DS, and SA at baseline and after 120 days. Sucrose (29–47%), raffinose (12–25%), galacturonic acid (7–18%), glucose (6–16%), and fructose (7–17%) were the most abundant, whereas rhamnose (1–6%) and galactose (1–3%) contributed the smallest fractions. These distributions agree with previous metabolomic evidence in *P. auritum*, which identified myo-inositol, fructose, galactose, and glucose [27], and with profiles reported for spinach and arugula, where sucrose and hexoses dominate alcohol-soluble fractions and raffinose ranks lower [19].

After 120 days, Figure 5A shows sucrose increased in CT, decreased in SA, and remained unchanged in DS. Accumulation of sucrose under water deficit is consistent with its role in lowering cellular osmotic potential and supporting water uptake. The decline in SA aligns with *Jacobea amequatica*, where SA application reduced sucrose and glucose (Figure 5D) and was associated with herbivory damage [38]. In cucumber seedlings, SA decreased sucrose in leaves but increased it in roots, while leaf glucose (Figure 5D) and fructose (Figure 5E) increased [39]. The increases in glucose and fructose observed here under SA agree with that mechanism and suggest enhanced synthesis and promotion of a redistribution of sucrose from leaves to other tissues (probably roots), along with accumulation of such hexoses in leaves [40].

Raffinose (Figure 5B) remained stable in DS and SA but declined in CT. Because raffinose is synthesized from sucrose (fructose + glucose) and galactose, stability under stress may indicate preservation of protective oligosaccharide pools. Stress-induced activation of raffinose synthase has been documented [41], with galactinol serving as donor of galactose which can be used either for raffinose synthesis or been accumulated as free galactose. The latter may be the case in *P. auritum* since the increase in galactose with DS and SA occurred without a clear increase in raffinose, which points to preferential retention of free galactose in leaves rather than incorporation into raffinose. The modest decrease in raffinose in CT, together with its relative stability in DS and SA, supports the view that stressed plants maintain oligosaccharides levels almost without variation, which may enhance the prebiotic effect of this plant in food preparations.

Galacturonic acid (Figure 5C) did not change in CT but decreased in DS and SA. Together with rhamnose, this monomer forms the backbone of rhamnogalacturonan-II (RG-II), a complex pectic domain important for wall architecture. Prolonged DS can compromise these domains, explaining the decline in free galacturonic acid. Rhamnose also decreased with DS, potentially reflecting diversion into glycosylation of anthocyanins via UDP-glycosyltransferases (e.g., UGT79B2, UGT79B3) known to be induced by abiotic stress in *Arabidopsis* [42]. The increases in total flavonoids in DS and SA observed in this work are compatible with such allocation. While SA-mediated regulation of sugars varies by species and growth conditions, the joint decrease in galacturonic acid and rhamnose under stress suggests wall remodeling that favors secondary metabolite formation at the expense of soluble pectic fragments.

Overall, Figure 5 indicates that DS conserves sucrose and increases free galactose while SA reduces sucrose and increases glucose and fructose, consistent with distinct allocation strategies: maintenance of compatible solutes under DS and enhanced translocation with local hexose accumulation under SA.

**Figure 6:**
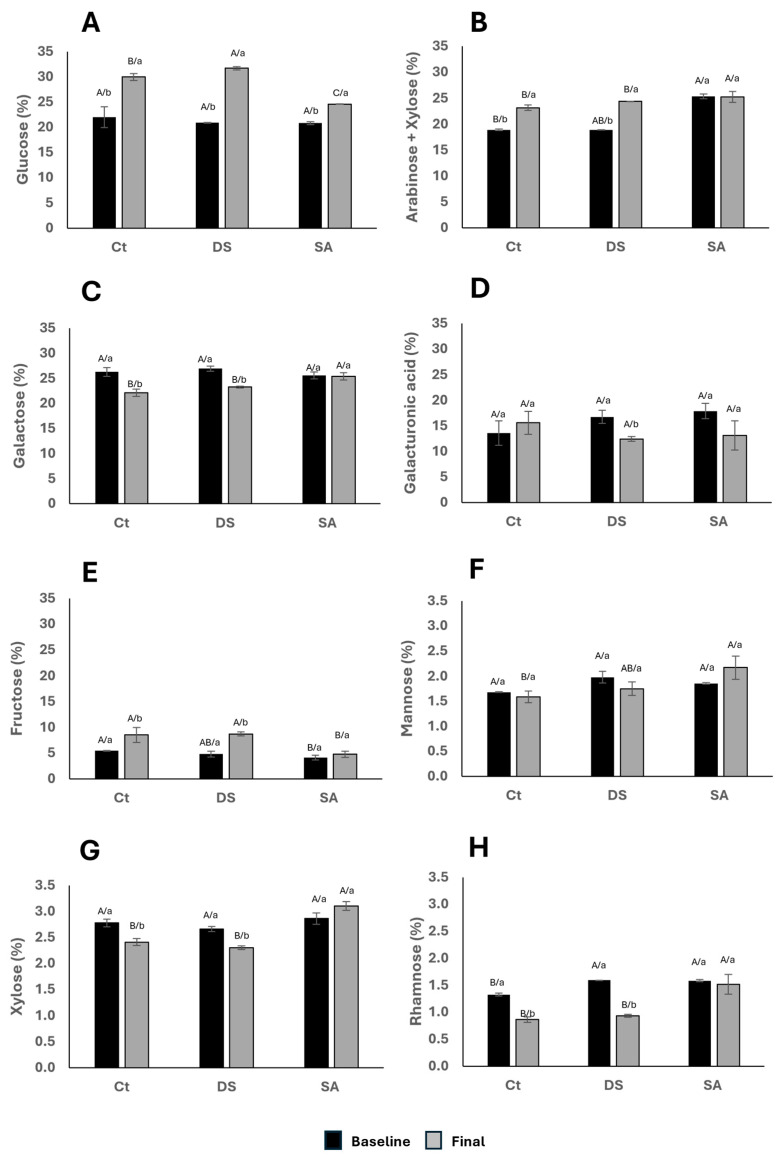
Monomeric composition presented from higher to lower abundance percentage of (**A**). Glucose; (**B**). Arabinose + Xylose; (**C**). Galactose; (**D**). Galacturonic acid; (**E**). Fructose; (**F**). Mannose; (**G**). Xylose; and (**H**). Rhamnose. Characters indicate comparisons between treatments (uppercase) and time (lowercase), respectively, using Tukey–Kramer test (*p* < 0.1) and t Student test (*p* < 0.1), respectively.

Acid hydrolysis followed by monosaccharide analysis provides the relative contribution of structural sugars. Figure 6 shows glucose (Figure 6A) as the most abundant monomer (22–32%), followed by galactose (22–27%) and arabinose + xylose (18–26%), with rhamnose at 0.5–1.7%. These proportions resemble those reported for spinach, alfalfa, and arugula, although *P. auritum* showed comparatively high glucose and galacturonic acid (Suarez et al., 2025) [19].

Glucose (Figure 6A) increased across all treatments after 120 days; DS ended with the highest values and SA the lowest. This pattern is consistent with the role of glucose in cellulose and hemicellulose biosynthesis. The relatively lower glucose under SA can be linked to growth inhibition through auxin-related regulatory pathways [41]. Arabinose + xylose (Figure 6B) increased in CT and DS but not in SA. These sugars are typical of arabinoxylans; their increase suggests reinforcement of hemicellulose networks in CT and underwater deficit, whereas SA appears to prioritize other metabolic sinks.

At baseline, galactose (Figure 6C) was similar among groups. After 120 days it decreased in CT and DS but was better maintained in SA, while galacturonic acid (Figure 6D) decreased significantly in DS. This contrast implies that drought affects pectic domains more than SA elicitation. Fructose (Figure 6E) was highest in CT at baseline and increased after treatment in CT and DS, with SA showing little change; this pattern is consistent with fructose functioning as an osmoprotectant and energy source during stress [43].

Mannose (Figure 6F) showed no baseline differences; after treatment it tended to be higher in SA (not significant), while CT and DS remained similar. Xylose (Figure 6G) showed no baseline differences but decreased in CT and DS and increased in SA, indicating that SA may favor specific hemicellulose remodeling. Rhamnose (Figure 6H) started higher in DS and SA than in CT and later declined in CT and DS, remaining comparatively stable in SA. The maintenance of rhamnose in SA is compatible with allocation to glycosylated phenolics rather than to soluble wall fragments.

Compared with glycomic databases [18], the galacturonic acid range detected here (10–16%) exceeds the 4–8% often reported for leafy greens, highlighting the contribution of pectins in *P. auritum*. Although mannose and rhamnose are minor components, they play structural roles; in *Arabidopsis* leaves, rhamnose is distributed among soluble fractions, water-soluble polymers, and matrix polysaccharides [44]. The slight reduction in xylose under DS agrees with the decrease in insoluble fiber after prolonged drought observed in this study, supporting the conclusion that water deficit weakens hemicellulose networks. In aggregate, Figure 6 supports stress-dependent reshaping of monosaccharide pools with consequences for wall architecture and precursor allocation to secondary pathways.

The variations in monosaccharide composition of *P. auritum* may influence its sensory contribution in traditional preparations. The higher glucose and fructose observed under CT and DS may contribute to the release of sweet notes or caramelization reactions during cooking, which may contribute to aroma and flavor along with essential oil components. Reduced galacturonic acid under DS, which reflects lower pectin content, could result in less softening upon heating, altering leaf tenderness in stews or wrapped dishes. Reinforcement of hemicellulose fractions (arabinose + xylose) may increase resistance to breakdown during cooking, while the relative stability of rhamnose under SA may sustain glycosylated phenolics that modulate bitterness or herbal notes. These observations suggest that stress-induced remodeling of carbohydrate pools has implications not only for wall structure but also for the texture and flavor released in culinary contexts. Given that *P. auritum* is mainly consumed in traditional cooked preparations, further research should integrate sensory analyses to determine how DS and SA induced changes in carbohydrate composition translate into modifications of aroma, flavor, and overall palatability.

### 3.5. In Vitro Digestion

**Figure 7:**
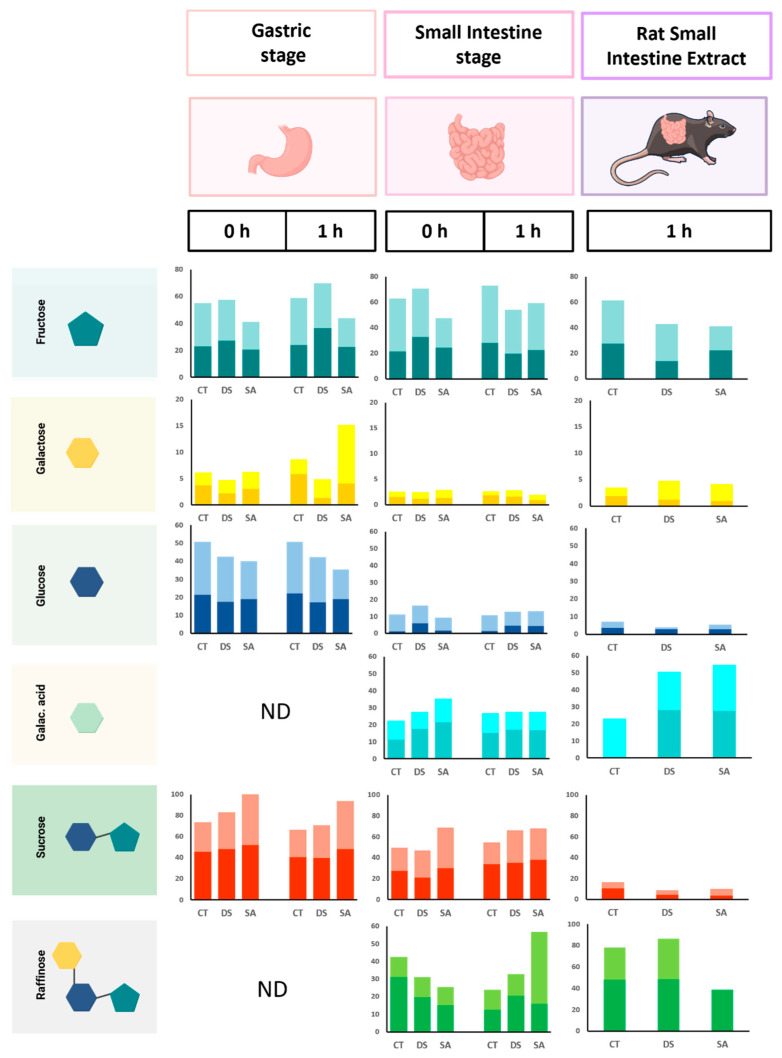
Relative abundance in percentage of low molecular weight carbohydrates (LMWCs) across gastric at 0 (G0) and 1 h (G1), intestinal at 0 (I0) and 1 h (I1), and RSIE stages for CT (control), DS (drought stress), and SA (salicylic acid). The lower, darker portion of each bar represents the baseline period, and the upper, lighter portion corresponds to the final period. Created in BioRender.com.

Figure 7 reports relative abundances of LMWC released during the gastric (G0, G1) and small-intestinal (SI0, SI1) phases of the INFOGEST protocol and after RSIE addition. The soluble fraction was analyzed because enzymatic digestion detaches sugars from the leaf matrix, allowing relative comparisons across stages and treatments.

Fructose maintained high proportions throughout digestion, indicating ready release from initial pools. SA showed lower fructose than CT and DS at G0 and G1, consistent with partial sequestration within intact tissues. During intestinal phases, fructose increased in the final period across all treatments, reflecting progressive release from the matrix. Within RSIE, DS started with the lowest fructose at baseline, while SA ended with the lowest value at the final time, indicating treatment-dependent hydrolysis dynamics (see Figure 7, fructose panels).

Galactose was highest in CT during G0–G1, while DS showed the lowest values at both periods. This distribution suggests that SA promotes accumulation of soluble galactose in extracellular sites rather than incorporation into structural domains after 120 days.

Under stress, soluble sugars can be redirected to apoplasts or vacuoles to stabilize osmotic conditions instead of contributing to wall biosynthesis [45] At SI0–SI1, galactose showed limited variation, with a modest increase at RSIE consistent with further release from pectin-rich structures by intestinal enzymes. Reports for tea polysaccharides also detected galactose, although less prominently in the intestinal stage than observed here [43].

Glucose followed a pattern similar to galactose during G0–G1 across treatments and increased at SI0–SI1, consistent with hydrolysis of oligosaccharides and starch by pancreatic α-amylase. A comparable retention-then-release pattern has been described for green tea leaves [46]. RSIE did not produce a marked increase in glucose, indicating limited liberation from bound forms at this final step, possibly due to enzyme–matrix interactions or partial inhibition by leaf constituents.

Galacturonic acid was absent at G0–G1 and detected only at SI0–SI1, confirming that its release depends on intestinal enzymes responsible for pectin degradation, and that pepsin activity and gastric acidity have negligible effects on this component. Additionally, its proportion increased at RSIE across groups, with CT showing the smallest initial values. This pattern suggests that DS and SA modify wall porosity and accessibility, facilitating later release of pectic fragments. Comparable observations in *Arabidopsis* link stress with altered pectin remodeling [45]. The RSIE-associated increase in galacturonic acid is consistent with homogalacturonan hydrolysis reported when intestinal extracts are included [16].

Sucrose peaked at G0–G1 among LMWC and remained notable at SI0–SI1 but decreased at RSIE for all treatments (see Figure 7, sucrose panels). This behavior is compatible with hydrolysis by sucrase–isomaltase, yielding fructose and glucose, as reflected in their increases at earlier stages. In matcha and green tea digestions, sucrose is often undetected during intestinal phases [46], an outcome reproduced here at RSIE.

Raffinose was absent at G0–G1 and appeared at SI0–SI1. SA showed a clear increase at SI1 in the final period, while at RSIE raffinose predominated in CT and DS (see Figure 7, raffinose panels). The persistence of raffinose at RSIE and its enrichment in CT and DS indicate resistance to small-intestinal hydrolysis and potential fermentation by colonic microbiota [47]. The higher raffinose at SI1 under SA suggests that elicitation modifies wall porosity and transport properties, facilitating earlier release before RSIE.

Leaf constituents can modulate digestive enzyme activity. Triterpene saponins from *P. auritum* inhibit α-amylase (23–76%) and α-glucosidase (10–65%) [48], which may limit glucose liberation during SI and RSIE and contribute to the modest glucose levels observed at late stages. In citrus pectin models, inclusion of RSIE doubled galactose and galacturonic acid release [16], consistent with the increases measured here for galacturonic acid at RSIE. Reported effects of SA on wall porosity [49] provide a plausible explanation for enhanced raffinose at SI1 in SA-treated leaves, increasing its availability for downstream fermentation and short-chain fatty acid formation.

These stage-dependent patterns of sugar release are not only of biochemical interest but may also influence human health. The moderation of glucose liberation by enzyme inhibition suggests a potential role in glycemic control, while the persistence of raffinose into RSIE indicates that it might reach colonic microbiota and may exert its prebiotic potential through colonic fermentation and short-chain fatty acid production. Increases in pectin-derived fragments such as galacturonic acid further highlight a possible contribution to gut barrier function and microbiota modulation, underscoring that abiotic stress in *P. auritum* may shape both digestibility and downstream physiological effects.

### 3.6. Antioxidant Activity

DPPH radical-scavenging activity showed no significant differences among treatments or time points, ranging 900–1200 μmol Trolox equivalents/100 g DW (Figure 4C). Although drought often enhances antioxidant capacity, the use of a single assay may underestimate differences. Rumpf et al. (2023) [50] compared assays for lignin and found DPPH to be the most variable, as it measures hydrogen atom transfer rather than single electron transfer. The essential oil components of *P. auritum*, mainly terpenes and safrole, lack hydroxyl groups and exhibit negligible activity, consistent with previous studies on isolated terpenes [51]. In addition, glycosylated flavonoids or those lacking C-ring double bonds show lower activity [52]. Our earlier analysis identified luteolin, apigenin, kaempferol, and cyanidin glycosides as dominant flavonoids [11] which may explain the limited changes in DPPH despite higher phenolic levels. Alizadeh et al. (2020) [32] reported a 42% rise in DPPH inhibition under drought in *S. hortensis*, underscoring species-specific responses.

In contrast, ABTS values increased ~50% after 120 days of DS (Figure 4D). Rumpf et al. (2023) [50] showed ABTS to be the least variable assay for lignin, and Samaniego Sánchez et al. (2007) [53] noted its strong correlation with TPC in virgin olive oil. The congruence between TPC (Figure 4A) and ABTS (Figure 4D) trends support this relationship. Conde-Hernández and Guerrero-Beltrán (2014) [33] measured antioxidant activity of dried *P. auritum* leaves at 15–20 mg Trolox equivalents/g DW, equivalent to 7492–9990 mmol/100 g DW, values higher than those in this study. Differences may stem from collection practices or extraction methods, even when protocols overlap. Overall, antioxidant activity was only significantly enhanced under DS when measured by ABTS, suggesting that phenolic enrichment translates to functional effects more clearly in this assay than in DPPH.

The increase in ABTS activity under drought stress coincides with an enrichment of phenolic compounds with higher reducing capacity. According to Apak et al. [52], phenolics exert antioxidant effects not only by scavenging model radicals but also by chelating transition metals and stabilizing oxidizable substrates, mechanisms that are more relevant in biological matrices. From a nutritional perspective, the observed increase suggests that drought-stressed *P. auritum* leaves could deliver a greater contribution to the dietary antioxidant pool, with potential implications for protecting lipids, proteins, and other cellular components from oxidative modification. Although absolute values were lower than those reported for dried leaves [33], the relative enhancement is within a range associated with improved functional properties in other plant foods [38].

## 4. Conclusions

Challenging environmental conditions present an adverse scenario for the biodiversity and continuity of traditional diets. Alterations in both nutritional and nutraceutical composition, as well as in the digestive release of such nutrients, represent an aspect that must increasingly be considered in future evaluations of edible plants. In this study, we assessed two abiotic stress scenarios: drought stress (DS) and salicylic acid (SA) treatment in *P. auritum*, a traditional quelite. DS was associated with reduced dietary fiber content and a probable redistribution of sugars between intracellular pools and structural components of the leaves, indicating changes in carbohydrate partitioning under water limitation. Galacturonic acid, rhamnose, and galactose content reflected the highest impact of DS. In contrast, SA exerted a greater influence on secondary metabolite synthesis, many of which are essential oil constituents in *P. auritum*, particularly oxygenated and hydrocarbon terpenes, as well as metabolites related to antioxidant responses under stress. To our knowledge, this is the first exploratory study addressing the relative abundance of low molecular weight carbohydrates (LMWC) in a *quelite* species. The analysis revealed that both DS and SA modulated the release of several LMWCs during simulated digestion, in some cases enhancing the relative abundance of specific sugars. These modifications may influence the potential prebiotic contribution of *P. auritum*, its role as a dietary source of functional compounds, and its adaptive strategies under environmental stress. Importantly, understanding how abiotic stressors shape the biochemical profile and nutrient release from this *quelite* offers mechanistic insight and practical guidance for safeguarding the nutritional quality and functional value of traditional edible plants. Such knowledge can inform conservation strategies, cultivation practices, and dietary recommendations that support the preservation of quelites and other traditional foods in the face of climate change.

## Figures and Tables

**Table 1:** Proximate composition, soluble, and insoluble fiber of *P. auritum* Kunth leaves after treatment of foliar application of salicylic acid and drought stress.

		Lipids	Protein	Ash	Carbohydrate	Soluble Fiber	Insoluble Fiber
Baseline	Ct	7.08 ± 0.80 A/a	1.25 ± 0.11 B/a	13.61 ± 0.29 B/b	78.05 ± 1.10 A/a	23.96 ± 0.90 B/b	41.78 ± 0.76 B/a
	SA	5.25 ± 1.92 A/a	1.90 ± 0.41 A/a	15.13 ± 0.80 A/a	77.72 ± 3.13 A/a	34.51 ± 1. 15 A/a	43.75 ± 0.79 B/a
	DS	6.88 ± 1.28 A/a	1.96 ± 0.45 A/a	16.44 ± 2.85 A/a	74.7± 1.13 A/b	20.53 ± 0.97 B/a	49.70 ± 1.69 A/a
Final	Ct	7.16 ± 1.32 A/a	1.58 ± 0.30 B/a	18.12 ± 0.19 A/a	73.21 ± 1.71 B/b	29.65 ± 4.31 A/a	35.04 ± 4.12 A/a
	SA	6.29 ± 1.79 A/a	1.59 ± 0.48 B/a	14.73 ± 0.22 B/a	77.40 ± 2.24 A/a	31.08 ± 2.25 A/a	41.92 ± 2.86 A/a
	DS	6.14 ± 0.44 A/a	2.09 ± 0.2 A/a	14.78 ± 0.20 B/a	76.98 ± 0.36 A/a	20.26 ± 1.43 B/a	42.95 ± 0.48 A/b

Results are expressed in percentage. Each result is the mean of 3 measurements ± standard deviation. Means of treatments were compared under Tukey–Kramer test (*p* < 0.05) (Uppercase) among treatments, and under *t*-Student test (*p* < 0.05) to compare final and baseline conditions (Lowercase) Ct: Control; SA: Salicylic acid; DS: Drought stress.

## Data Availability

The original contributions presented in the study are included in the article. Further inquiries can be directed to the corresponding author.

## Supplementary Materials

The following supporting information can be downloaded at https://www.mdpi.com/article/10.3390/foods14203543/s1, Figure S1: Certificate of the taxonomic identification of *Piper auritum* Kunth; Figure S2: VIP score plot from PLS-DA models highlighting the most discriminant metabolites (VIP > 1.0).
