# Supplementary material for: Abiotic Stress Alters the Nutritional, Metabolomic, and Glycomic Profiles of Piper auritum Kunth
Source: Foods. 2025 Oct 17;14(20):3543. doi: 10.3390/foods14203543 (PMC12563271; doi:10.3390/foods14203543)
Supplement: Supplementary file 1 [file foods-14-03543-s001.zip › foods-3904421-supplementary.pdf]

Supplementary Material

Abiotic Stress Alters the Nutritional, Metabolomic, and Glycomic Profiles of *Piper auritum*

Adriana Chico Peralta a, Mar Villamiel b, Paola Isabel Angulo-Bejarano a, Aurea K. Ramírez-Jiménez a.

[a] Tecnologico de Monterrey, School of Engineering and Science, Av. Eugenio Garza Sada 2501 Sur, Monterrey, NL, C.P. 64849, Mexico.

[b] Grupo de Química y Funcionalidad de Carbohidratos y Derivados, Instituto de Investigación en Ciencias de la Alimentación (CIAL) (CSIC-UAM) CEI (CSIC + UAM), Nicolás Cabrera, 9, Campus de la Universidad Autónoma de Madrid, 28049 Madrid, Spain

Correspondence to: aramirezj@tec.mx

UNIVERSIDAD AUTÓNOMA DE QUERÉTARO
FACULTAD DE
CIENCIAS NATURALES

Santiago de Querétaro, Querétaro, a 20 de febrero de 2024

A QUIEN CORRESPONDA:

La identificación solicitada por el Dra. Aurea Ramírez Jiménez., corresponde a la especie ***Piper auritum*** **Kunth, familia PIPERACEAE.** El ejemplar ingresará a nuestra colección con el número QMEX00006865

.

Se expide la determinación para los fines que se estime conveniente.

ATENTAMENTE

Dr. Luis G. Hernández Sandoval
Curador del Herbario "Dr. Jerzy Rzedowski" QMEX
Tel. (442) 192 12 00 Ext. 5319, 5324

UNIVERSIDAD AUTÓNOMA DE QUERÉTARO
Domicilio: Av. de las Ciencias s/n; Juriquilla, Delegación Santa Rosa Jáuregui, C.P. 76230, Querétaro, Qro. Tels: (442) 192-12-00, Directo, 5301, Investigación y posgrado, 5371, Coordinaciones: Biología, Horticultura Ambiental, Medicina Veterinaria y Zootecnia y Nutrición; 5310, 5311, Coordinaciones: Geografía Ambiental y Microbiología, 65214

SOMOS UAQ
EDUCAR CRECER CONSOLIDAR

Figure S1. Certificate of the taxonomic identification of Piper auritum Kunth. The identification was performed in the ethnobotanical collection in the "Dr. Jerzy Rzedowski", at Universidad Autónoma de Querétaro, México.

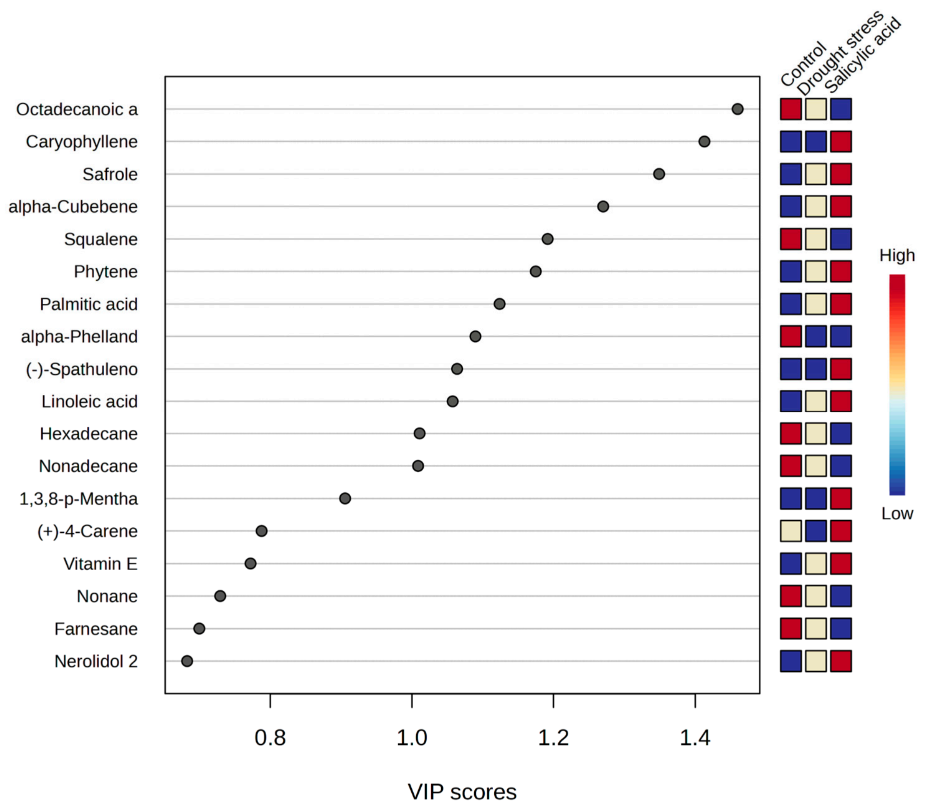

Figure S2. VIP score plot from PLS-DA models highlighting the most discriminant metabolites (VIP > 1.0).
